# In Vivo and In Vitro Anticancer Activity of Doxorubicin-loaded DNA-AuNP Nanocarrier for the Ovarian Cancer Treatment

**DOI:** 10.3390/cancers12030634

**Published:** 2020-03-09

**Authors:** Chang-Seuk Lee, Tae Wan Kim, Da Eun Oh, Su Ok Bae, Jaesung Ryu, Hyejeong Kong, Hyeji Jeon, Hee Kyung Seo, Seob Jeon, Tae Hyun Kim

**Affiliations:** 1 Department of Chemistry, Soonchunhyang University, Asan 31538, Korea; eriklee0329@sch.ac.kr (C.-S.L.); dani0607@sch.ac.kr (D.E.O.); bsovj@sch.ac.kr (S.O.B.); 2 Department of Medical Life Science, Soonchunhyang University, Asan 31538, Korea; ktwdreem@naver.com (T.W.K.); rjs652@naver.com (J.R.); angelkonghj@gmail.com (H.K.); 3 Department of Obstetrics and Gynecology, College of Medicine, Soonchunhyang University Cheonan Hospital, Cheonan 31151, Korea; hjjeon@schmc.ac.kr (H.J.); sweet115747@gmail.com (H.K.S.)

**Keywords:** Nanomedicine, doxorubicin, gold nanoparticles, ovarian cancer, drug delivery

## Abstract

In this study, we have determined the anticancer activity of doxorubicin (Dox)-loaded DNA/gold nanoparticle (AuNP) nanocarrier (Dox-DNA-AuNP) for the treatment of ovarian cancer. The anticancer effect of Dox-DNA-AuNP was evaluated in vitro using the EZ-Cytox cell viability assay on three human ovarian cancer cell lines, SK-OV-3, HEY A8, and A2780. Dox-DNA-AuNP exhibited outstanding activity with good IC_50_ values of 4.8, 7.4, and 7.6 nM for SK-OV-3, HEY A8, and A2780, respectively. In vivo evaluation further demonstrated the superior anticancer effects of Dox-DNA-AuNP by inhibiting tumor growth compared to free Dox in an established SK-OV-3 xenograft mice model. Dox-DNA-AuNP showed about a 2.5 times higher tumor growth inhibition rate than free Dox. Furthermore, the immunohistochemical analysis of Ki67 antigen expression showed the lowest number of proliferative cells in the ovarian tumor tissue treated with Dox-DNA-AuNP. These results suggest Dox-DNA-AuNP might be a potential effective agent in ovarian cancer chemotherapy.

## 1. Introduction

Ovarian cancer is the leading cause of death and third most common gynecological cancer in women of developed countries [1]. More than 75 % of women have advanced disease (International Federation of Gynecology and Obstetrics (FIGO) stage IIIC or IV) at diagnosis, of whom a substantial proportion is unwell and unfit and have a 5-year survival rate of less than 30 % [2]. Most ovarian cancer patients undergo cytoreductive surgery followed by adjuvant chemotherapy (i.e., platinum/paclitaxel-combination chemotherapy), as there may be some cancer cells still in the body. Chemotherapy is useful to kill small amounts of tumor cells that may still exist after surgery, or to shrink large tumors to make surgery easier. While approximately 75 % of patients initially respond to the chemotherapy, most of them relapse with chemoresistance which results in treatment failure and causes over 90 % of deaths [3]. Second-line chemotherapies are often required, however, in contrast to first-line chemotherapy, the anticancer effect of second-line chemotherapies is usually bad.

Conventional chemotherapy has limitations with a lack of solubility in an aqueous solution, side effects at high-doses, and a short bloodstream circulation half-life [4,5,6]. To overcome these limitations, many approaches using nanoparticles for the drug delivery carrier, such as liposomes [7,8,9], dendrimers [10,11], silicon nanoparticles (SiNPs) [12,13], and gold nanoparticles (AuNPs) [14,15,16,17] have been proposed. Among those nanoparticles, the AuNPs have some advantages as the drug delivery carrier, such as that it is easy to synthesize with controlled size and shape, simple surface chemistry, and high biocompatibility [18,19]. Furthermore, AuNPs can provide a high surface-area-per-volume ratio which leads to more anticancer drugs using AuNPs than naïve drugs. Therefore, an AuNP-based nanocarrier can reduce the dose-dependent side effect of anticancer chemotherapy [20,21].

Doxorubicin (Dox) is one of the most commonly used anticancer drugs for ovarian cancer chemotherapy that is approved by the Food and Drug Administration (FDA) of the United States. In particular, the initial treatment of Dox is reported to improve the overall survival rate for the ovarian cancer patients in whom first-line platinum-based treatment has failed [22]. However, there is still no systematic research on the in vivo anticancer activity of various Dox formulations for ovarian cancer treatment. Due to its intrinsic fluorescence and structural properties, Dox has been widely utilized for drug delivery studies as an imaging agent and anticancer drug in combination with nanodrug carriers such as inorganic nanoparticles [23,24,25], dendrimers [26,27,28], and graphene oxide [29,30], etc [31,32,33,34].

**Scheme 1:**
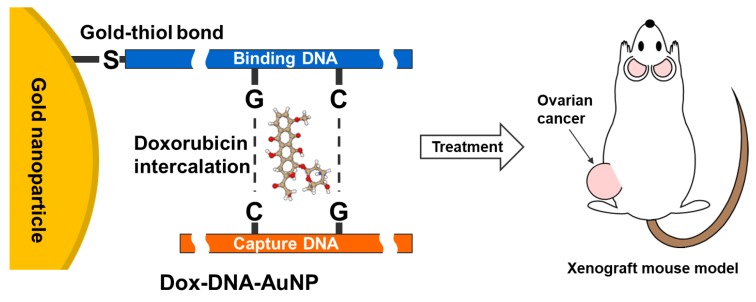
Schematic diagrams of the Dox-DNA-AuNP for the ovarian cancer treatment.

Herein, we determined the in vivo and in vitro anticancer activity of Dox-loaded DNA/AuNP (Dox-DNA-AuNP) for ovarian cancer treatment. To the best of our knowledge, this is the first extensive study on the assessment of in vitro and in vivo anticancer activity of the proposed nanodrug system for ovarian cancer treatment. Scheme 1 illustrates the structure and workflow of Dox-DNA-AuNP, which was prepared using AuNP conjugated with guanine (G)-cytosine (C) rich DNAs into which Dox was loaded through intercalation. The anticancer effect of Dox-DNA-AuNP was examined in the human ovarian cancer cell lines SK-OV-3, HEY A8, and A2780 in vitro using a EZ-Cytox cell viability assay. In vivo, the inhibition ratio of tumor growth in treated-to-control (T/C) tumors was also examined in ovarian cancer-bearing mice models. The results show that Dox-DNA-AuNP has an excellent anticancer activity for the human ovarian cancer, which holds a great promise to use it as an alternative for platinum/paclitaxel-combination chemotherapy to treat the patients.

## 2. Results and Discussion

### 2.1. Characterization of DOX-DNA-AuNPs

**Figure 1:**
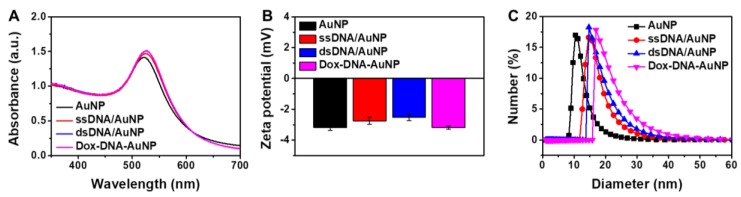
The characterization of the Dox-DNA-AuNP using (**A**) UV-vis spectroscopy, (**B**) zeta potential, and (**C**) dynamic light scattering.

In the preparation of AuNP-based nanomedicine, the size consistency of AuNPs in each preparation step is an important parameter to control the effective drug loading into the carrier. Because of its intrinsic self-aggregation property, as time goes on, the size of AuNPs is speculated to increase along with the possibility of crystallization [35]. Therefore, the size of the AuNPs in each synthesis step should be monitored. Figure 1A shows the UV-vis spectra of nanoparticles at each step during the preparation of Dox-DNA-AuNP.

All nanoparticle samples exhibited the same absorption shape with an absorption peak around 520 nm, which corresponds to the AuNP diameter of 13 nm [36]. This indicates the presence of consistent 13 nm-sized AuNPs and no aggregation of AuNPs during the preparation process of Dox-DNA-AuNP. This result is presumably due to the electrostatic repulsive forces of the nanoparticles from their negatively charged surfaces. To confirm the negative charged surfaces of the AuNPs, zeta potential measurements were employed to evaluate the surface charge through different steps of the preparation process. As shown in Figure 1B, the surface charges of nanoparticles at each step of the synthesis remained negative. Through the synthesis steps for the Dox-DNA-AuNP, the surface modification was changed to negatively charged DNAs from citrate anions, so that the surface charge of the AuNPs remained negative. Dynamic light scattering (DLS) analysis was performed to identify the preparation process of the Dox-DNA-AuNP by comparing the hydrodynamic size of the AuNPs during each step of the procedure (Figure 1C). After the treatment of binding DNA (single stranded DNA, ssDNA), the hydrodynamic diameter increased from 11 nm to 15 nm, suggesting the successful immobilization of ssDNA on AuNP. The sequential introduction of capture DNA caused a further slight enhancement to 16 nm, revealing hybridization of the capture DNA to ssDNA/AuNP, forming double stranded DNA (dsDNA)/AuNP. After subsequent loading of Dox, the nanoparticle size also increased, presumably due to the intercalation of Dox into the G-C rich region of dsDNA on AuNP.

### 2.2. In Vitro Release Test of Dox from Dox-DNA-AuNP

**Figure 2:**
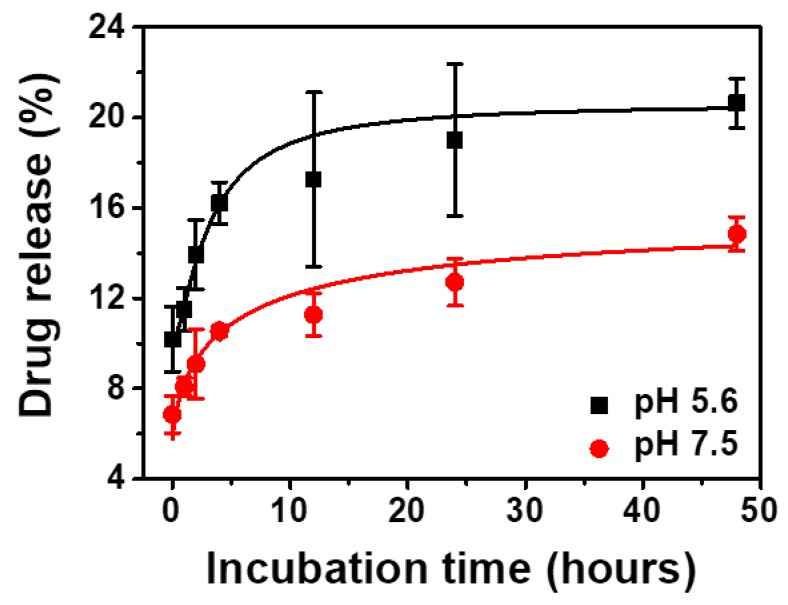
In vitro drug release test of Dox-DNA-AuNP depending on the time and pH conditions.

The stability of the drug delivery carrier is an important factor to achieve good efficacy of drug delivery systems. The stability of Dox-DNA-AuNP was evaluated indirectly by examining the in vitro time-dependent drug release of Dox from Dox-DNA-AuNP. Figure 2 shows the in vitro release profiles of Dox from the Dox-DNA-AuNP in RPMI 1640 media with pH 5.6 and pH 7.5 at 36.5 ˚C for 48 h.

Prior to the drug release test, the drug loading content (w/w%) and drug entrapment efficiency (%) of the Dox-DNA-AuNP were calculated according to following equation and found to be 3.0% and 81%, respectively.

(1) $$\mathrm{Drug}\;\mathrm{loading}\;\mathrm{content}\;\left(w/w\%\right)=\frac{weight\;of\;loaded\;DOX}{weight\;of\;DOX\;loaded\;DNA-AuNP}\times 100\;$$

(2) $$\mathrm{Drug}\;\mathrm{entrapment}\;\mathrm{efficiency}\;\left(\%\right)=\frac{weight\;of\;DOX\;in\;DNA-AuNP}{weight\;of\;DOX\;fed\;initially}\times 100\;$$

As shown in Figure 2, an initial-burst release was observed within 5 h in both buffers, although the amount of Dox in pH 5.6 released twice as high as in pH 7.4. Up to 48 h thereafter, the Dox release slowly increased and saturated at approximately 14.6% in the pH 7.5 buffer. This indicates about 85% loading of Dox on Dox-DNA-AuNP, suggesting the high stability of Dox-DNA-AuNP. From this result, it can be inferred that Dox-DNA-AuNP has ample stability in the systemic circulation to diffuse and get to the target organ, because of the similarity between pH 7.5 RPMI and the blood condition. Meanwhile, the Dox-DNA-AuNPs showed a 1.42 times higher drug release rate in pH 5.6 RPMI for 48 h. This is ascribed to the pH-dependent structural transformation of the DNA that contains the Dox. In the acidic condition, the G and C bases undergo depurination, leading to the structural transformation of the dsDNA in which intercalated Dox can be released. This result represents the possibility of the pH-dependent cancer-targeting drug delivery of the Dox-DNA-AuNP, because of the acidic condition of the cancer microenvironment [37,38].

### 2.3. In Vitro Time-Dependent Cellular Uptake of Dox-DNA-AuNP

**Figure 3:**
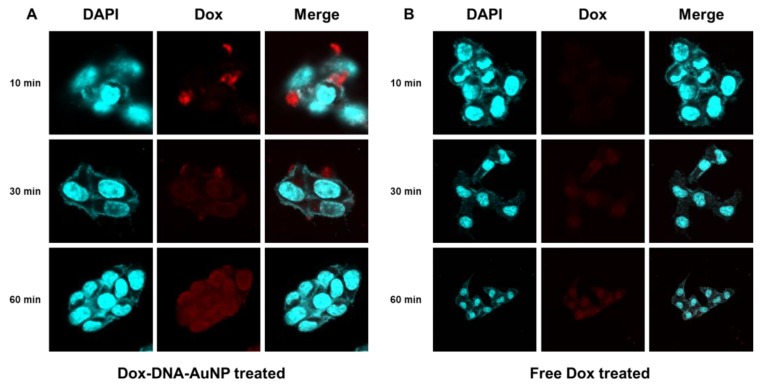
Cellular uptake test of (**A**) Dox-DNA-AuNP, and (**B**) free Dox for the SK-OV-3 cell with different incubation time. Dox concentration is same (10 µM) for Dox-DNA-AuNP and free Dox. Fluorescence images were obtained by confocal microscopy. Cyan color represents DAPI stained nuclei observed at 440–480 nm with the 358 nm excitation; red color represents the fluorescence of Dox observed at 500–700 nm with the 480 nm excitation.

To evaluate the cellular internalization of Dox-DNA-AuNP, its intracellular uptake efficiency was investigated in SK-OV-3, HEY A8, and A2780 ovarian cancer cell lines by confocal microscopy. Figure 3 shows the confocal microscopy images of the SK-OV-3 cell lines after treatment with Dox-DNA-AuNP and free Dox for 10, 30, and 60 min.

With increasing time of treatment, the SK-OV-3 cells emitted increased red fluorescence of Dox, which overlaps with the blue fluorescence from DAPI. This indicates the colocalization of Dox in the nucleus, and also demonstrates that the Dox released from Dox-DNA-AuNP could reach the nuclei of tumor cells, possibly by certain enzymatic reactions such as nucleases. Meanwhile, unlike Dox-DNA-AuNP, free Dox-treated SK-OV-3 cells emitted a faint red fluorescence (Figure 3B). This indicates that the cellular permeability of free Dox was limited, which however was enhanced through the conjugation in the form of Dox-DNA-AuNP to gain access into cells. Video S1 (Supplementary Materials) shows the time-dependent cellular uptake of the Dox-DNA-AuNP to SK-OV-3 cells with different incubation times and camera focus (depth). The camera focus moved from the top of the SK-OV-3 cells to the bottom. At 10 min after drug treatment, the red fluorescence was condensed at the top of the cells, representing the attachment of Dox-DNA-AuNP to the SK-OV-3 cells. At 30 min, the red fluorescence of Dox moved to the middle of the cells with a circular form, indicating the endosome containing the Dox-DNA-AuNP. Lastly, at 60 min, the red fluorescence of Dox was dispersed to the nuclei of the SK-OV-3 cells in the same region of DAPI stained. This demonstrates that the Dox was released from Dox-DNA-AuNP and successfully reached to the nuclei of the SK-OV-3 cells. Similar results were observed for HEY A8, and A2780 cancer cell lines in the confocal microscopy analysis in Figures S1 and S2 (Supplementary Materials), respectively. These results represent the higher drug delivery efficacy of Dox-DNA-AuNPs than free-Dox treatment. Quantitative analysis of cellular uptake was also performed using fluorescence spectroscopy with the treated cell lysates from the SK-OV-3, HEY A8, and A2780 cell lines (Figure S3). With increasing time of treatment with Dox-DNA-AuNP, the Dox concentration in all three cell lysates increased, while the free Dox concentration remained almost the same. This result is in good agreement with the cellular uptake test in Figure 3.

### 2.4. In Vitro Cytotoxicity of Dox-DNA-AuNP Against Ovarian Cancer Cells

**Figure 4:**
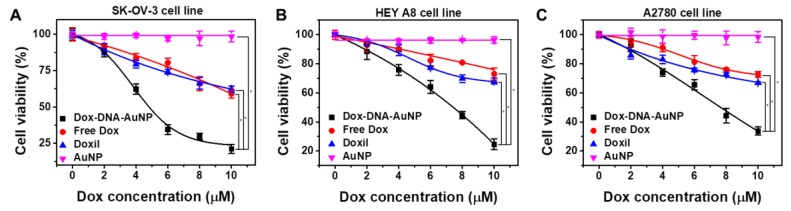
In vitro cytotoxicity studies of Dox-DNA-AuNP. Cell viabilities of (**A**) SK-OV-3 cell line, (**B**) HEY A8 cell line, and (**C**) A2780 cell lines after treatment with the different concentration of free Dox, Doxil^®^, AuNP and Dox-DNA-AuNP. Concentrations of AuNP are same with those of AuNP in Dox-DNA-AuNP. Statistical comparisons were made using *t-*test; * *p* < 0.001

To investigate the anticancer effect of the Dox-DNA-AuNP, the in vitro inhibition efficacy of Dox-DNA-AuNP and free Dox against ovarian cancer cells was evaluated through studying the cell viability of SK-OV-3, HEY A8, and A2780 cells incubated with different Dox formulations. Figure 4 shows the cell viabilities of the SK-OV-3, HEY A8, and A2780 cell lines after treatment with an equivalent concentration of Dox-DNA-AuNP, free Dox, and Doxil^®^.

It was found that Dox-DNA-AuNP, free Dox, and Doxil^®^ exhibited a cytotoxic effect on ovarian cancer cells in a concentration-dependent manner. However, Dox-DNA-AuNP showed significantly higher cytotoxicity against all of three cell lines than free Dox at the same range of Dox concentration. Interestingly, no significant anticancer effect of AuNP was observed, confirming the good anticancer effect of the proposed formulation design of nanoparticles. Accordingly, it is inferred that Dox-DNA-AuNP could remarkably enhance the anticancer effect on ovarian cancer cells. For the quantitative anticancer effect of Dox-DNA-AuNP, the half-maximal inhibitory concentration (IC_50_) was calculated in the ovarian cancer cell lines to be 3.8 μM, 5.9 μM, and 6.1 μM for SK-OV-3, HEY A8, and A2780, respectively in terms of Dox concentration. These can be converted into the concentration values of Dox-DNA-AuNP, which are 4.8 nM, 7.4 nM, and 7.6 nM for SK-OV-3, HEY A8, and A2780, respectively.

### 2.5. In Vivo Anticancer Activity of Dox-DNA-AuNP

**Figure 5:**
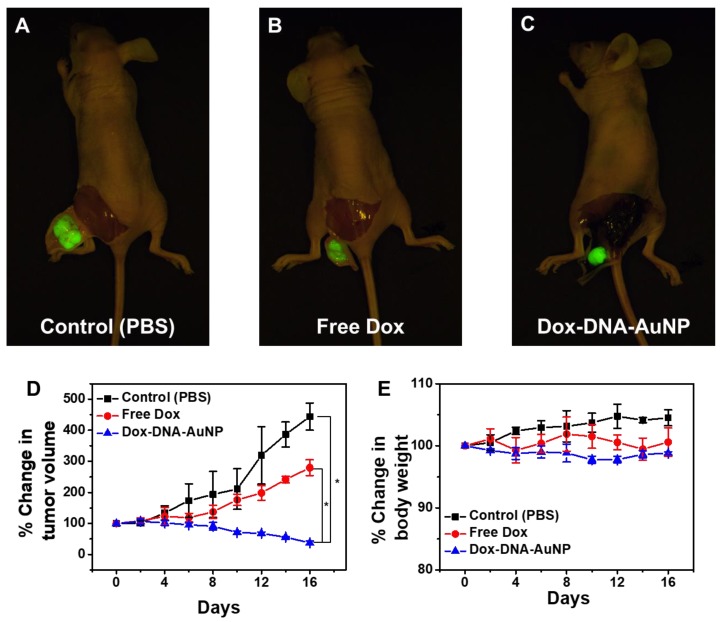
The merged images of optical and fluorescent images obtained from the xenografted SK-OV-3-tumor bearing mice model treated with (**A**) PBS buffer, (**B**) free Dox, and (**C**) Dox-DNA-AuNP at the end of the test. SK-OV-3 tumor contains green fluorescence proteins (GFP). The time dependent changes in the (**D**) tumor volumes and (**E**) body weights in each drug treated tumor bearing mice model. Statistical comparisons were made using *t-*test; * *p* < 0.001

Given the satisfactory anticancer effect of Dox-DNA-AuNP in vitro, we finally evaluated the potential of Dox-DNA-AuNP to promote an in vivo anticancer activity. Toward this end, Dox-DNA-AuNP, free Dox, and the PBS buffer as a control group were injected into mice bearing SK-OV-3 xenograft tumors which contained green fluorescent proteins (GFP). Figure 5A–C shows the fluorescence images of the tumor-bearing mice that merged with optical images.

The green fluorescence was well observed from GFP, representing the xenografted tumors in all three groups. Dox-DNA-AuNP exhibited a significant reduction in tumor size (Figure 5C) at the end of the test, compared to the PBS buffer (Figure 5A) and free Dox (Figure 5B), although initial sizes of tumors in all three groups were comparable. Figure 5D clearly shows a more drastic suppressive effect of the Dox-DNA-AuNP on tumor growth than the free Dox and PBS buffer. While final tumor sizes increased to 444% and 279% in the PBS-treated and free Dox-treated groups, respectively, the size of the Dox-DNA-AuNP-treated tumor decreased to 37.39% from day 0. The tumor growth inhibition rate, which was calculated based on tumor volume change, of free Dox and Dox-DNA-AuNP is 37.07% and 91.58% (about 2.5 times higher), respectively. As shown in Figure 5E, the body weights of the tumor-bearing mice were also monitored while showing negligible changes on all experimental days. These results indicate the high tumor growth inhibition efficacy and low toxicity of the Dox-DNA-AuNP in vivo.

**Figure 6:**
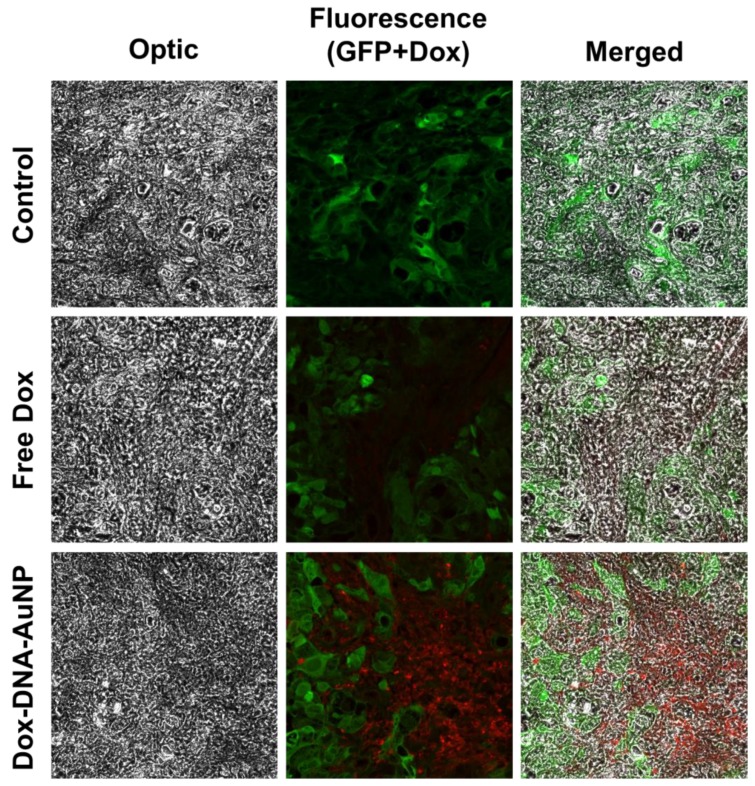
Confocal microscopy images of distribution of control (PBS), free Dox, and Dox-DNA-AuNP in tumor tissue excised from tumor-bearing mouse after the end of the treatment. Green color represents GFP contained in tumor cells observed at 440–480 nm with the 395 nm excitation; red color represents the fluorescence of Dox observed at 500–700 nm with the 480 nm excitation.

After the drug treatments of the mice, we also examined the biodistribution effect of Dox-DNA-AuNP on tumor tissue and other organs excised from the xenografted SK-OV-3-tumor bearing mice. Detection of Dox, as monitored by confocal microscopy, indicated substantial differences in the distribution of Dox-DNA-AuNP for tumor (Figure 6) vs. other organs (Figure S4). As shown in Figure 6, the red fluorescence of Dox from free Dox and Dox-DNA-AuNP were observed throughout tumor tissue containing GFP. On the contrary, there was no significant fluorescence shown in other organs (Figure S4). This indicates no accumulation of Dox-DNA-AuNP in other organs and its successful distribution on tumor sites, thereby anticipating negligible side effects.

### 2.6. In Vivo Immunohistochemical Study

**Figure 7:**
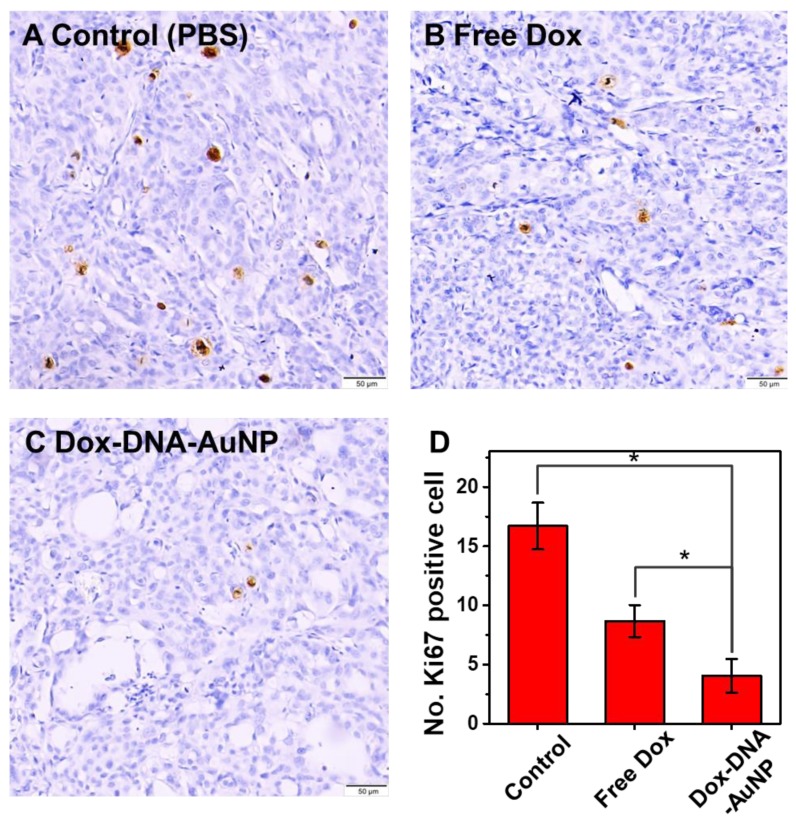
Immunohistochemical study for the Ki67 expression by (**A**) control (PBS buffer treated), (**B**) free Dox, and (**C**) Dox-DNA-AuNPs treated tumors. (**D**) The quantitative analysis of IHC results. Statistical comparisons were made using *t-*test; * *p* < 0.001.

In order to evaluate the in vivo proliferative capacity of cancer cells after treatment, the immunohistochemistry (IHC) of Ki67 expression was performed on the tumor sections. Ki67 is a widely used cellular marker for cell proliferation, presenting during all active phases of the cell cycle including G1, S, G2, and M stages. High expression of Ki67 is associated with tumor aggression, vascular invasion, tumor metastasis, and poor response to chemotherapy [39]. Figure 7 shows the IHC studies for Ki67 stained by using paraffin-embedded xenograft cancer tissue blocks of the mice with (A) control (PBS-treated) group, (B) Dox-treated group, and (C) Dox-DNA-AuNP-treated group. For analyzing the results of IHC, we randomly fixed five square zones (400 μm × 400 μm) in each sectioned slide. As shown in Figure 7D, tumors treated with the Dox-DNA-AuNP had a significantly lower Ki-67 labeling index (4.04 ± 0.71), compared to the free Dox (8.68 ± 0.67)-treated or control (16.96 ± 0.88) groups, demonstrating the effective tumor growth inhibition of Dox-DNA-AuNP. This result indicates Dox-DNA-AuNP plays a key role in enhancing the inhibition of tumor cell proliferation, induction of tumor cell apoptosis, and shows the potential to support tumor suppression [40,41].

## 3. Materials and Methods

### 3.1. Materials

Gold(III) chloride trihydrate (99.9% trace metals basis), phosphate buffer saline (PBS, pH 7.4), sodium citrate dihydrate, doxorubicin hydrochloride, fetal bovine serum, Roswell Park Memorial Institute (RPMI) 1640 media, and Fluoroshield^TM^ with 4′,6-diamidino-2-phenylindole (DAPI, histology mounting medium) were purchased from Sigma-Aldrich. Thiol terminated binding DNA (5′-HS-CC-AAAAAAAAAA-TCG-TCG-TCG-TCG-TCG-TCG-TCG-3′) and capture DNA (5′-CGA-CGA-CGA-CGA-CGA-CGA-CGA-3′) were synthesized by GenoTech Co., (Daejeon, Korea).

### 3.2. Measurements

The particle size and surface charge of AuNPs were measured by the UV-Vis spectrophotometer S-3100 (SCINCO Co., Ltd. Seoul, Korea), and the Zetasizer Nano ZS-90 DLS instrument (Malvern, UK). For the drug-loading test, the fluorescence intensity of Dox was monitored upon the addition of AuNP, binding DNA (ssDNA), dsDNA, ssDNA-AuNP, and dsDNA-AuNP. The fluorescence intensity of Dox was measured at excitation and emission wavelengths of 480 and 520 nm, respectively. The drug release test was performed with the thermo-shaker; Dox-DNA-AuNP were incubated in 36.5 °C with 350 rpm shacking. After the incubation, the released Dox was collected by taking the supernatant of Dox-DNA-AuNP. The fluorescence spectroscopy with FS-2 (SCINCO Co., Ltd. Seoul, Korea) was performed to measure the concentrations of Dox.

### 3.3. Synthesis of Dox-DNA-AuNP

Dox-DNA-AuNP was synthesized similar to the procedure previously reported by us [16]. There were three steps for the preparation of Dox-DNA-AuNPs; (i) synthesis of AuNPs, (ii) surface modification of AuNPs with the binding DNA, and (iii) Dox loading to the DNA-AuNP. Briefly, 10 mL of 1 mM HAuCl_4_ was heated until boiling temperature with stirring. When the solution boiled vigorously, 1 mL of 38.8 mm sodium citrate was added quickly. The reaction was stopped when the color of the solution changed from dark blue to burgundy. Then the AuNP solution was cooled down to room temperature. For immobilization of the DNA, 100 μL of 17 μM binding DNA was added to 1 mL of 10 nm AuNP. The mixed solution was incubated at the shacking incubator with 350 rpm at 25 °C for 16 h. After that, 100 μL of 1 M sodium chloride and 100 μL of 0.1 M sodium phosphate buffer were added to the solution. After 24 h, the mixed solution was centrifugated twice at 13500 rpm for 20 min to discard the extra binding DNA. For the hybridization of binding DNA and capture DNA, 100 μL of 17 μM capture DNA was added to the previous mixed solution with 350 rpm at 95 °C for 5 min, followed by incubation at a shacking incubator with 350 rpm at 25 °C for 1 h. Then, the DNA-AuNP was purified with centrifugation twice at 13500 rpm for 20 min. Finally, for the Dox loading to the DNA-AuNP, 100 μL of 100 μM Dox was added to 1 mL of DNA-AuNP solution and the solution was incubated in a shacking incubator with 350 rpm at 25 °C for 1 h, followed by purification with centrifugation twice at 13500 rpm for 20 min. The drug loading capacity of Dox-DNA-AuNP was measured quantitatively by fluorescence spectroscopy. Initially, the fluorescence of a free Dox (500 nM) was measured at a range of 500-700 nm, with the excitation maxima at 480 nm, and then the fluorescence quenching was monitored with increasing concentrations of DNA-AuNP. As the amount of DNA-AuNP increased, the fluorescence intensity of the Dox solution decreased quantitatively and ultimately reached a maximum value of quenching. This decrease in fluorescence intensity is due to the intercalation of Dox into DNA leading to quenching of fluorescence of the bound Dox because of photoelectron transfer (PET) from DNA. On incubation with DNA-AuNP, maximal quenching of the Dox fluorescence was observed at approximately 810 molar equivalence of Dox to DNA-AuNP, which is in agreement with the predicted presence of CG sequence binding sites.

### 3.4. Cell Cultures

SK-OV-3, HEY A8, and A2780 ovarian cancer cell lines were obtained from the Korean Cell Line Bank (Seoul, Korea). The cells were grown in RPMI 1640 media containing 10 % FBS and 1 % ABS at 37 °C in a humid atmosphere containing 5 % CO_2_. GFP was transfected into SK-OV-3 cell line by using lentiviruses. The ovarian cancer cells were cultured by common cancer cell culture procedures [42].

### 3.5. In Vitro Uptake Test

For the cellular uptake of Dox-DNA-AuNP, SK-OV-3, HEY A8, and A2780 cells were incubated in Dox-DNA-AuNP solution with different incubation times, and were stained by Fluoroshield^TM^ with DAPI for 10 min. Then, the samples were washed twice with PBS solution. The cellular uptake images were obtained by confocal microscopy FV-10i (Olympus Co., Ltd. Tokyo, Japan).

### 3.6. In Vitro Cytotoxicity Test

Cytotoxicity assays were performed as previously reported [16], with minor modifications. SK-OV-3, HEY A8, and A2780 cells were seeded at a density of 1 × 10^4^ cells per well in 96-well cell culture plates. The cell viability test was performed by EZ-Cytox cell viability assay kit. To evaluate the effect of Dox-DNA-AuNP on SK-OV-3, HEY A8, and A2780 cell viability, 200 μL of free Dox and Dox-DNA-AuNP solution at different concentrations (0, 2, 4, 6, 8, and 10 μM) were added to each well containing cells and incubated at 37 °C for 24 h. The cells were washed with PBS to remove the unbound free Dox and Dox-DNA-AuNP. The percent viability of cells was determined as the percent color formation in experimental wells relative to the control well. Each EZ-Cytox cell viability assay was performed in triplicate.

### 3.7. In Vivo Anticancer Effect

In vivo analyses were performed as previously reported [16], with minor modifications. For the tumor-bearing mice model, BALB/c nude mice (6-week-old) were purchased from Korea Orient bio, Inc. (Sungnam-si, Korea) and maintained under conventional housing conditions. Ovarian cancer xenograft studies were performed to compare the effect of PBS, free Dox, and Dox-DNA-AuNP on tumorigenesis. GFP tagged SK-OV-3 Ovarian cells (1 × 10^6^ cells/200 μL) were subcutaneously inoculated into the flanks of female BALB/c nude mice to generate xenografts. Three groups of mice were subcutaneously administered with 10 mm each of Dox-DNA-AuNPs, free Dox, and PBS respectively on the 0, 2, 4, 6, 8, 10, 12, 14, and 16th day. The drug was treated when the tumor volume came to 0.52 μm^3^. Mice were sacrificed at the end of the treatment. The size of the tumor was measured using a digital caliper and the tumor volume was determined with the following formula: length (mm) × width (mm) × height (mm) × 0.52. All the animal experiments were conducted in accordance with the Guidelines of the Soonchunhyang University Institutional Animal Care and Use Committee (IACUC, approval # SCH18-0055) on 9 September 2019.

### 3.8. Immunohistochemistry

Paraffin-embedded xenograft cancer tissue blocks were sectioned at 5 μm thickness. Antigen retrieval was performed by heating the sectioned slides in 10 mM citrate buffer in a pressure-cooker for 15 min. After heating, the sectioned slides were cooled at room temperature for 1 h. The sectioned slides were then incubated overnight at 4 °C with human Ki67 1st antibody (1:200, Abcam, Cambridge, MA). The sectioned slides were then incubated at room temperature with price goat anti-rabbit 2nd antibody (1:1000, Invitrogen, Carlsbad, Germany) for 1 h, followed by treatment with 100 μL 3′-3-diaminobenzide (Dako). For analyzing the result of IHC, we randomly fixed five square zones (400 μm × 400 μm) in each sectioned slide to measure the number of dyed cells and averaged them. Then we conducted a statistical analysis using the Student t-test. The statistical analysis was performed using SPSS 19.0 (SPSS, Chicago, IL, USA).

### 3.9. Statistical Analysis

The study t-test was used to statistically analyze differences in the expression of Ki67 genes when IHC was applied to the xenograft cancer tissue taken from mice treated with control, Dox, and Dox-DNA-AuNP. To compare the IHC results, the Student t-test was introduced. Statistical analysis was performed using SPSS 19.0 (SPSS, Chicago, IL, USA) for Windows OS and *p* < 0.05 was considered significant.

## 4. Conclusions

We demonstrated in vitro and in vivo anticancer activity of the Dox-DNA-AuNP drug delivery system for ovarian cancer treatment. The synthesized Dox-DNA-AuNP was characterized with UV-vis spectroscopy, zeta potential, dynamic light scattering, and fluorescence spectroscopy. Dox-DNA-AuNP showed a significant stability with pH 7.5 for 48 h, while observing only 14.85% of drug releases. In the acidic condition, Dox-DNA-AuNP released drugs 1.42 times higher than pH 7.5, representing the possibility of the pH-dependent drug release system; due to the acidic condition of the tumor microenvironment, Dox-DNA-AuNP might be good drug delivery carrier for cancer treatment. In vitro proliferation test confirmed the better anticancer effect of Dox-DNA-AuNP than free Dox, with good IC_50_ values of 4.8, 7.4, and 7.6 nm for SK-OV-3, HEY A8, and A2780, respectively. The cancer growth inhibition of Dox-DNA-AuNP was also confirmed in vivo with the xenograft mice model over 16 days of treatments. Dox-DNA-AuNP had about a 2.5 times higher tumor growth inhibition rate than free Dox, indicating good cancer growth inhibition. Furthermore, the in vivo anticancer effect of Dox-DNA-AuNP was assessed with IHC analyses, showing decreases in the number of Ki67-expressed tumor cell, indicating suppressed tumor proliferation by Dox-DNA-AuNP. Taken together, the results demonstrate that Dox-DNA-AuNP can be an effective therapeutic agent for the human ovarian cancer treatment. Further study is ongoing to increase the efficient delivery of the nanodrug system based on Dox-DNA-AuNP by endowing its active targeting ability to tumor sites.

## Supplementary Materials

The following are available online at https://www.mdpi.com/2072-6694/12/3/634/s1, Figure S1: Cellular uptake test of (A) Dox-DNA-AuNP, and (B) free Dox for the HEY A8 cell with different incubation time, Figure S2: Cellular uptake test of (A) Dox-DNA-AuNP, and (B) free Dox for the A2780 cell with different incubation time, Figure S3: (A) Standard curve of free Dox obtained from standard solution of free Dox in RPMI 1640 media. Quantitative analysis of cellular uptake of free Dox and Dox-DNA-AuNP in (B) SK-OV-3, (C) HEY A8, and (D) A2780 cell lines upon the treatment of Dox-DNA-AuNP and free Dox with different incubation time of 10, 30, and 60 min. After treatment of Dox-DNA-AuNP and free Dox, the cell lysates were obtained by 30 min of ultrasonication and 15000 g, 15 min of centrifugation. The fluorescence intensity of cell lysates was measured by fluorescence spectroscopy at 500–700 nm of emission with 480 excitation wavelengths, Figure S4. Optical and fluorescence images of organs excised from tumor-bearing mouse after the end of the treatment, Video S1: Time-dependent cellular uptake of the Dox-DNA-AuNP to SK-OV-3 cells with different incubation time and camera focus (depth).
