# Supplementary material for: In Vivo and In Vitro Anticancer Activity of Doxorubicin-loaded DNA-AuNP Nanocarrier for the Ovarian Cancer Treatment
Source: Cancers (Basel). 2020 Mar 9;12(3):634. doi: 10.3390/cancers12030634 (PMC7139456; doi:10.3390/cancers12030634)
Supplement: Supplementary file 1 [file cancers-12-00634-s001.zip › Supplementary Materials_rev.docx]

Supplementary Materials: In vivo and in vitro anticancer activity of doxorubicin-loaded DNA-AuNP nanocarrier for the ovarian cancer treatment

Chang-Seuk Lee, Tae Wan Kim, Da Eun Oh, Su Ok Bae, Jaesung Ryu, Hyejeong Kong, Hyeji Jeon, Hee Kyung Seo, Seob Jeon and Tae Hyun Kim


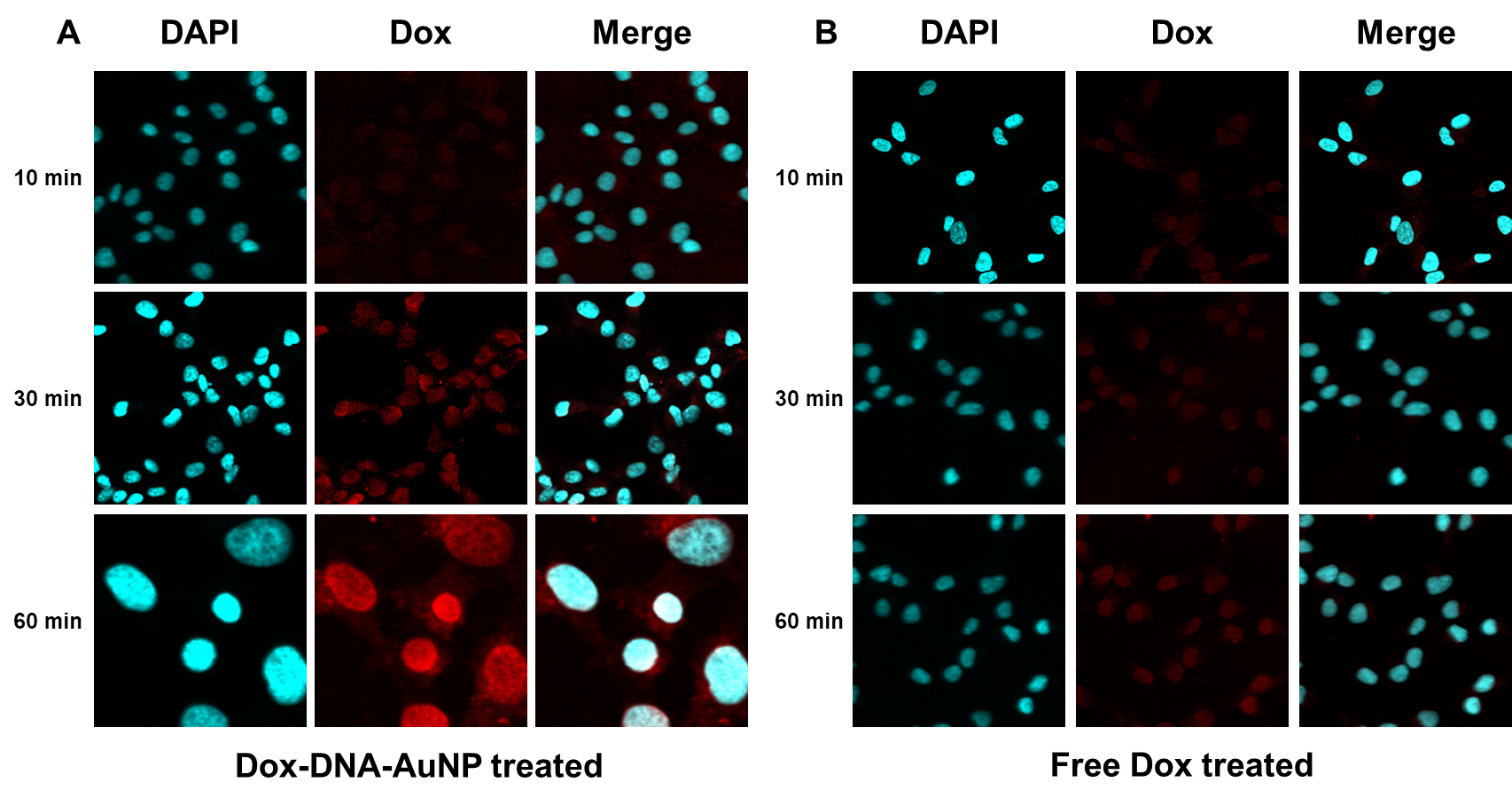


**Figure S1.** Cellular uptake test of (A) Dox-DNA-AuNP, and (B) free Dox for the HEY A8 cell with different incubation time. Dox concentration is same (10 µM) for Dox-DNA-AuNP and free Dox. Fluorescence images were obtained by confocal microscopy. Cyan color represents DAPI stained nuclei observed at 440-480 nm with the 358 nm excitation; red color represents the fluorescence of Dox observed at 500-700 nm with the 480 nm excitation.


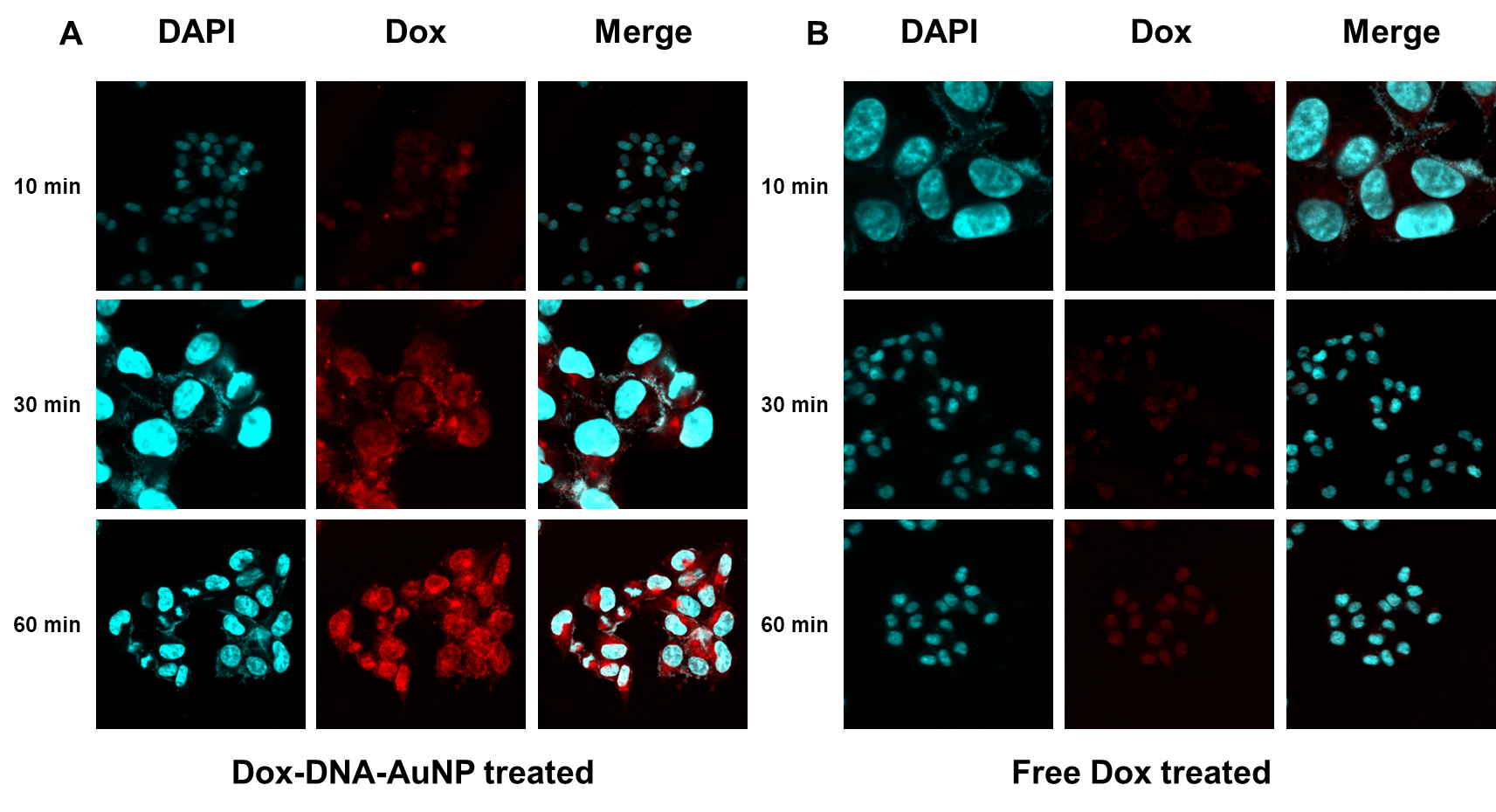


**Figure S2.** Cellular uptake test of (A) Dox-DNA-AuNP, and (B) free Dox for the A2780 cell with different incubation time. Dox concentration is same (10 µM) for Dox-DNA-AuNP and free Dox. Fluorescence images were obtained by confocal microscopy. Cyan color represents DAPI stained nuclei observed at 440-480 nm with the 358 nm excitation; red color represents the fluorescence of Dox observed at 500-700 nm with the 480 nm excitation.


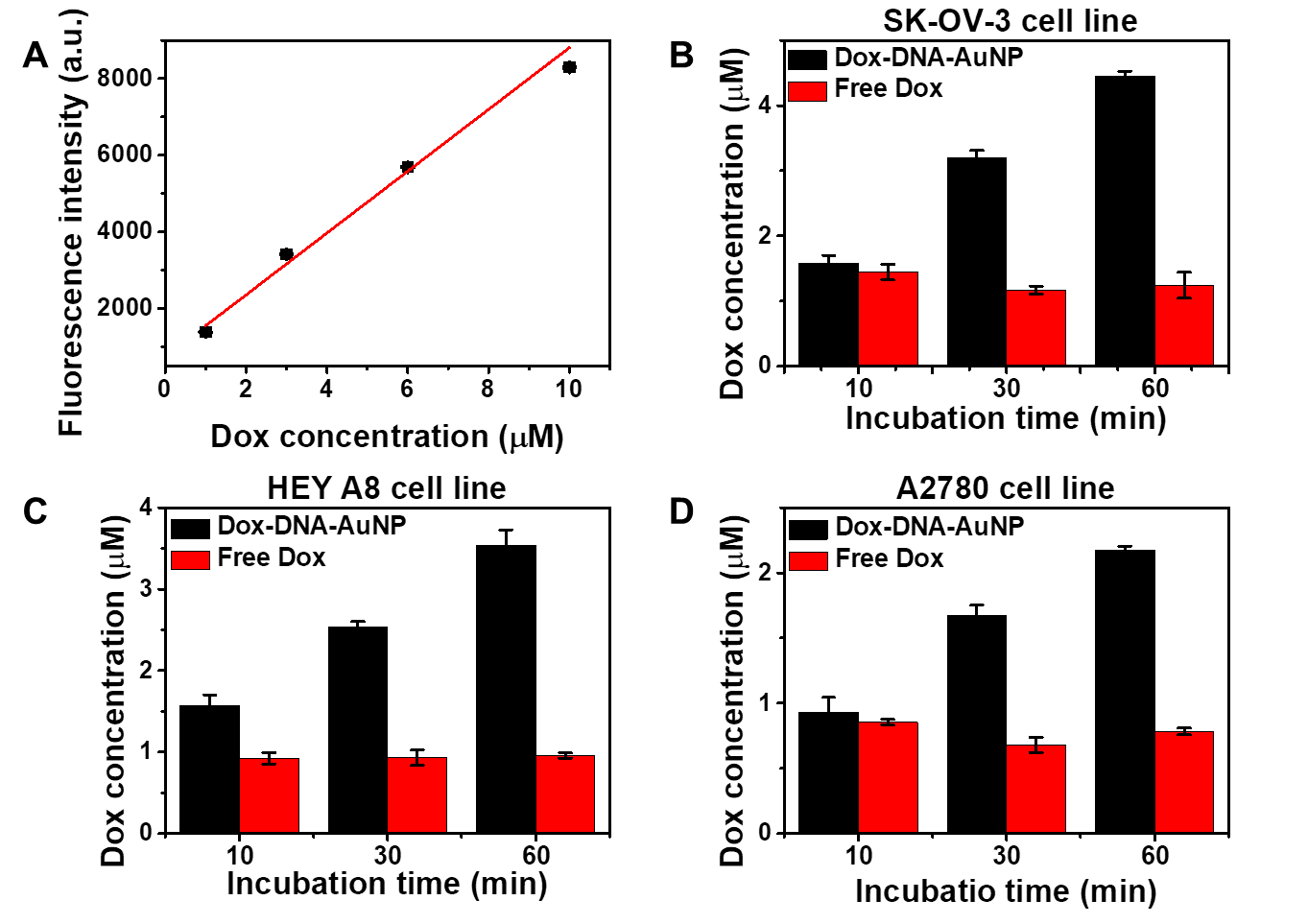


**Figure S3.** (A) Standard curve of free Dox obtained from standard solution of free Dox in RPMI 1640 media. Quantitative analysis of cellular uptake of free Dox and Dox-DNA-AuNP in (B) SK-OV-3, (C) HEY A8, and (D) A2780 cell lines upon the treatment of Dox-DNA-AuNP and free Dox with different incubation time of 10, 30, and 60 min. After treatment of Dox-DNA-AuNP and free Dox, the cell lysates were obtained by 30 min of ultrasonication and 15000 g, 15 min of centrifugation. The fluorescence intensity of cell lysates was measured by fluorescence spectroscopy at 500-700 nm of emission with 480 excitation wavelengths.


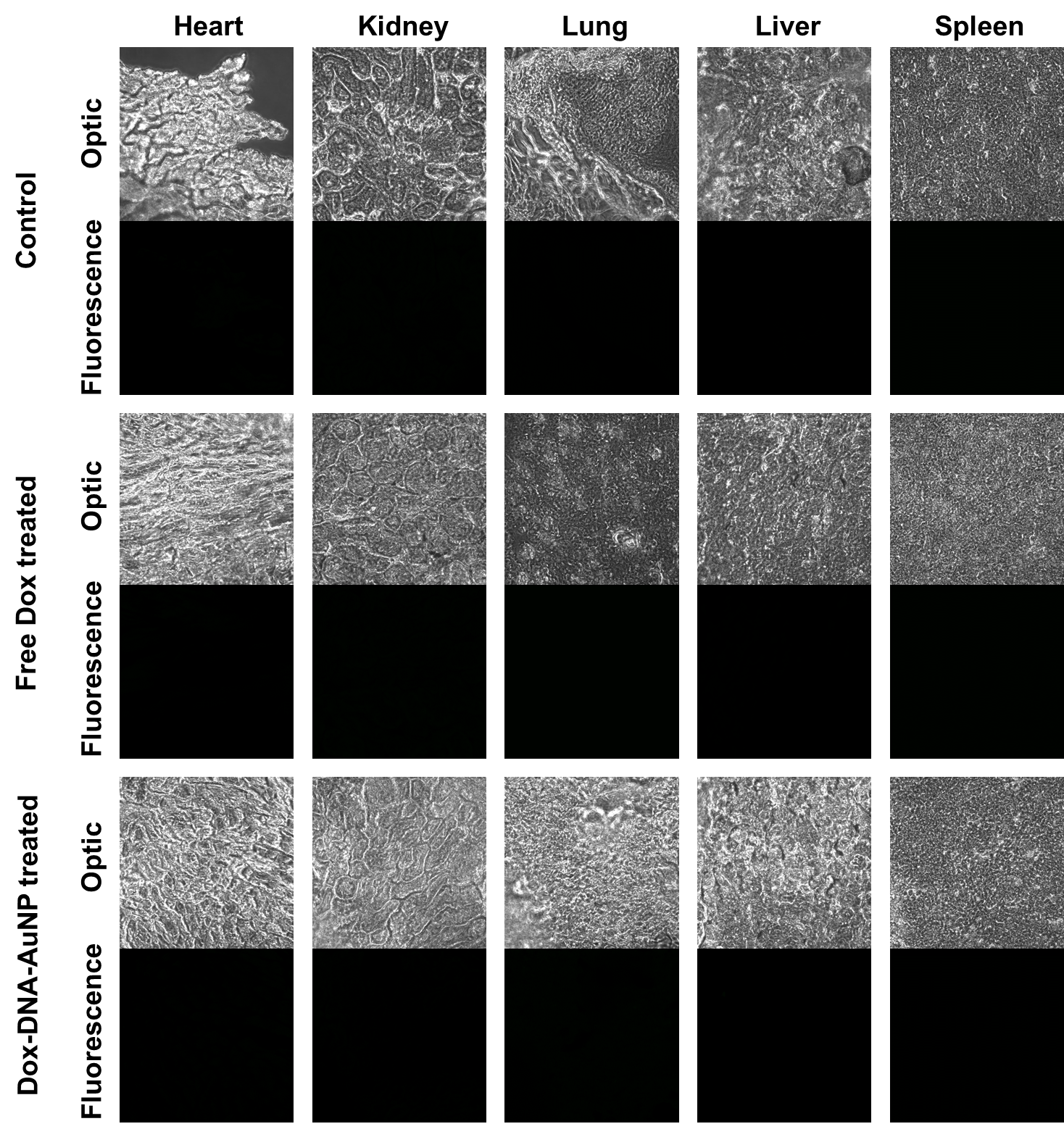


**Figure S4.** Optical and fluorescence images of organs excised from tumor-bearing mouse after the end of the treatment.
